# Fetal Rhesus D Genotyping and Sex Determination from Maternal Plasma of Rhesus D-Negative Antenatal Population: The Usefulness of Conventional Polymerase Chain Reaction in Resource-limited Settings

**DOI:** 10.1155/2020/4913793

**Published:** 2020-10-16

**Authors:** Otchere Addai-Mensah, Edward Y. Afriyie, Samuel Asamoah Sakyi, Christian Obirikorang, Max Efui Annani-Akollor, Eddie-Williams Owiredu, Francis A. Amponsah, Richard Vikpebah Duneeh, Evans Asamoah Adu

**Affiliations:** ^1^Department of Medical Diagnostics, Faculty of Allied Health Sciences, College of Health Sciences, Kwame Nkrumah University of Science and Technology, Kumasi, Ghana; ^2^Department of Molecular Medicine, School of Medical Science, Kwame Nkrumah University of Science and Technology, Kumasi, Ghana; ^3^Department of Medical Laboratory Sciences, School of Allied Health Sciences, University of Health and Allied Sciences, Ho, Ghana

## Abstract

**Background:**

This prospective cohort study evaluated the usefulness of conventional PCR in genotyping fetal Rhesus D (RhD) and sex from the maternal plasma of RhD-negative (RhD−) antenatal population in resource-limited settings.

**Methods:**

Thirty apparently healthy RhD− pregnant women with RhD positive (RhD+) partners were included. Blood samples were collected from each participant (in the third trimester of pregnancy) for DNA extraction/purification and fetal RhD genotyping.

**Results:**

Out of the 30 samples, 26 (86.7%) were found to be RhD+ while 4 (13.3%) were RhD−. The RhD+ comprised 24 (80.0%) RhD+ based on exons 5, 7, and 10 combined. Exons 5 and 7 were detected in two additional samples but not exon 10. Serological phenotyping of neonatal blood confirmed 26 RhD+ and 4 RhD−. There was a perfect agreement between the fetal RhD genotype and neonatal RhD phenotyping after delivery for exons 5 and 7 (concordance = 100%, *κ* = 100.0%, diagnostic accuracy = 100%, *p* < 0.0001) while exon 10 presented with an almost perfect agreement (concordance = 93.3%, *κ* = 76.2%, diagnostic accuracy = 93.3%, *p* < 0.0001). Regarding the prenatal test for the SRY gene, 9 (30.0%) were predicted to be males and the remaining 21 (60.0%) were females. All the 9 and 21 anticipated males and females, respectively, were confirmed after delivery (concordance = 100%, *κ* = 100.0%, diagnostic accuracy = 100%).

**Conclusion:**

Our study suggests that conventional PCR using the SRY, RhD exons 5 and 7 could be useful for predicting fetal sex and RhD from maternal peripheral blood in resource-limited settings.

## 1. Background

The Rhesus (Rh) blood group system is one of the most polymorphic and immunogenic systems known in humans. In the past decade, extensive investigations have yielded considerable knowledge about the molecular background of this system [1]. The antigens of the Rh (RhAg) blood group system, namely, D, C, c, E, and e are encoded by the homologous genes *RHD* and *RHCE*, both located on chromosome 1 [1].

An RhD-negative phenotype primarily occurs as a result of the deletion of the entire *RHD* gene. Rh antibodies (RhAb) are formed due to the exposure of an Rh-negative mother to cells which bears the RhAg, commonly through transfusions or pregnancy [2, 3]. These Abs have been implicated in Hemolytic Transfusion Reactions, Autoimmune Hemolytic Anemia, and Hemolytic Disease of the Fetus and Newborn (HDFN) [4]. HDFN is an important cause of perinatal mortality and morbidity arising from transplacental passage of maternal RhD Ab, mostly IgG, their binding to fetal erythrocyte RhD Ag, and the subsequent destruction of the fetal erythrocytes [5, 6]. This underscored the need for preemptive strategies against fetal haemolysis and subsequent fetal loss.

The introduction of anti-D prophylaxis has drastically reduced the global incidence of RhD immunization from 14% to 0.8–1.5%, with some countries having as low as 0.2–0.4% [7]. Nonetheless, the use of anti-D prophylaxis is not without risks (such as infections) as Rh immunoglobulin is derived from pooled human plasma [3, 8]. There is thus the need for effort intensification to avoid unessential exposure through the confirmation of fetal RhD status.

Advances in RhD genotyping technology have made it possible to noninvasively determine fetal RhD from analysis of circulating cell-free fetal DNA (ccffDNA) in maternal plasma as against amniocentesis [6, 9, 10]. ccffDNA originates from apoptotic macrovesicles separated from trophoblast cells of the placenta [11, 12]. It circulates in maternal blood at low levels detectable from the 7^th^ week of gestation—its level increases proportionally with increasing gestational age until it diminishes and disappears 1-2 days after delivery [13]. The advent of robotic systems of fetal DNA isolation has also made it possible for high-throughput testing [14–16]. However, the cost involved in using these systems may be too huge for most developing countries to defray. Recently, the use of real-time polymerase chain reaction (qPCR) has gained considerable attention in its usefulness in ascertaining fetal sex and RhD genotype [17–19]. Nevertheless, this method is not only expensive and requires relatively high expertise but also unavailable in most developing countries.

Unlike developed countries where prenatal RhD genotyping is considered routine and prophylaxis given only after genotype is determined, many developing countries embrace the practice of administering anti-D immunoglobulin prophylaxis to all RhD-negative pregnant women with RhD positive husbands. Rationalization for this practice can be ascribed to the possibility of bearing an RhD positive fetus as well as the lack of sophisticated equipment to prenatally determine fetal RhD status. There is however an indication that a significant proportion of RhD-negative women carry RhD-negative fetus [20]; thus, there is a possibility that anti-D immunoglobulin could be administered superfluously to a substantial number of women in such instances.

This highlights the need for developing countries to determine fetal RhD genotype to ensure that anti-D immunoglobulin is purposefully administered to the right candidate [21, 22]. Additional merit for fetal RhD genotyping is that it precludes the cost burden of anti-D immunoglobulin as well as the risks associated with the administration of blood products [10, 23].

This study evaluated the usefulness of the comparatively less expensive conventional PCR for fetal sex and RhD genotyping at a teaching hospital in Kumasi, Ghana. The findings of this study will equip policymakers with the necessary information that would inform the inclusion of fetal RhD genotyping as routine testing during pregnancy in developing countries with limited resources.

## 2. Methods

### 2.1. Study Design and Participants

This was a prospective cohort study conducted at the Komfo Anokye Teaching Hospital (KATH), Kumasi, Ghana. The duration of the study was one year: between January 2017 and 2018. Thirty (30) apparently healthy RhD-negative pregnant women with RhD positive partners attending the antenatal clinic were purposively recruited. Only participants who consented after the study objectives were explained to them were included in the study.

### 2.2. Ethical Considerations

Ethical approval for this study was obtained from the Committee on Human Research, Publications and Ethics (CHRPE), School of Medical Sciences, Kwame Nkrumah University of Science & Technology (Ref: CHRPE/RC/252/17). Written informed consent was obtained from all participants who opted to participate. Participation was voluntary, and respondents were assured that the information obtained was strictly for research and academic purposes only and were guaranteed the liberty to opt out of the study at their own convenience.

### 2.3. Sample Collection and Preparation

Five milliliters (5 ml) of venous blood was collected from each participant into EDTA tubes and spun at 2000 g for 10 minutes to obtain the plasma which was transferred into fresh tubes using filter tips and recentrifuged at 3000 g for 10 minutes. The supernatant was separated and stored at −80°C until further processing. All samples were processed within 6 hours of sample collection.

### 2.4. DNA Extraction/Purification and Genotyping

DNA was extracted using the standard modified inorganic (salting out) method as previously described by Miller et al. [24]. Briefly, cell lysis was performed by suspending the sample in lysis buffer (155 mM NH_4_Cl, 10 mM NH_4_CO_3_, 1 mM EDTA), followed by incubation on ice and centrifugation at 3,000 rpm and 4°C for 30 min. The pellets were resuspended in TE buffer and centrifuged to obtain white blood cell pellets which were resuspended in nucleic lysis buffer (400 mM NaCl, 10 mM Tris-HCl, 2 mM Na_2_-EDTA, pH 8.2). Cell lysates were digested with proteinase K and 10% sodium dodecyl sulfate (SDS) followed by the addition of 6 M NaCl and nucleic lysis buffer and centrifugation at 4,600 rpm for 60 min. DNA precipitation was performed by the addition of cold 96% ethanol. The quantities of the isolated DNA were measured using the NanoDrop 3300 Fluorospectrometer (Thermo Fisher Scientific Inc., USA) and A260/280  of 1.8–2.0 was used to define DNA purity (Supplemental Material: Figure S1). Each sample was assessed for fetal RhD and sex genotype by conventional PCR as previously described [25]. Briefly, thirty cycles of denaturation for 1 min at 92°C, primer annealing for 1 min at 49°C, and primer extension for 1 min at 72°C were performed, followed by a final extension for 9 min at 72°C. The reaction products were separated by agarose gel electrophoresis (3% agarose gel with 0.5 *μ*g/ml of ethidium bromide) and visualized under ultraviolet light (Supplemental Material: Figure S2). In an effort to enhance the specificity of the assay, we included three RHD gene regions, namely, RhD exons 5, 7, and 10. ccffDNA was confirmed using Y chromosome specific primer SRY. The primers for fetal SRY, RhD exons 5, 7, and 10 are shown in Supplemental Material: Table S1.

### 2.5. Confirmation of the Fetal Sex and RhD Status

Sex neonates were ascertained at birth. Furthermore, peripheral blood samples were collected immediately after birth from each neonate and RhD phenotype determined based on serological blood grouping (Diahem Diagnostics, Switzerland).

### 2.6. Statistical Analysis

Continuous and categorical data were presented as median (interquartile range) and frequency (percentages), respectively. Chi-square test and Cohen's Kappa were used to assess the association and concordance between prenatal and postnatal measures. A *p* value <0.05 was considered statistically significant. Statistical analysis was performed using IBM SPSS version 25.0.

## 3. Results

The average maternal and gestational ages of the study participants were 28.0 years and 33 weeks, respectively. A higher proportion were in their third trimester (80.0%), were married (73.3%), and had secondary education (53.3%) (Table 1).

Out of the 30 maternal samples analyzed for fetal RhD genotype, 26 (86.7%) were detected as RhD positive while 4 (13.3%) were detected as RhD-negative. The RhD positives comprised a total of 24 (80.0%) RhD positives based on RhD exons 5, 7, and 10 combined. RhD exons 5 and 7 were detected in two additional samples (2/6.6%) but not RhD (exon 10) (Table 2). Serological phenotyping of neonatal blood confirmed 26 RhD positives (86.7%) and 4 (13.3%) RhD negatives. There was perfect agreement (concordance = 100%, *κ* = 100.0%, diagnostic accuracy = 100%) between fetal RhD genotyping and neonatal RhD phenotyping after delivery for exons 5 and 7 while exon 10 presented with almost perfect agreement (concordance = 93.3%, *κ* = 76.2%, diagnostic accuracy = 93.3%) (Table 3).

Regarding the prenatal test of the SRY gene, 9 (30.0%) of the cases were predicted to be males and the remaining 21 (60.0%) females (Table 2). All 9 and 21 anticipated males and females, respectively, were confirmed after delivery (concordance = 100%, *κ* = 100.0%, diagnostic accuracy = 100%) (Table 3).

## 4. Discussion

Most developed countries perform prenatal RhD genotyping and prophylaxis is given only after fetal RhD positive genotype in an RhD-negative mother is determined. The robotic systems [14–16] as well as qPCR are utilized in these countries for highly accurate fetal RhD genotyping [17, 18]. However, these methods are not only expensive but also unavailable in most developing countries. For this reason, many healthcare facilities in developing countries embrace the practice of administering anti-D immunoglobulin prophylaxis to all RhD-negative pregnant women with RhD positive partners irrespective of fetus RhD status. Though useful as there have been drastic reductions in the incidence of RhD immunization since the inception of the anti-D immunoglobulin prophylaxis, the disadvantages associated with unsighted administration of the prophylaxis underpin the need for prenatal fetal RhD genotyping. This study evaluated the expediency of the less expensive conventional PCR for fetal sex and RhD genotyping in resource-limited settings.

Of the 30 maternal samples investigated, 26 (86.7%) were RhD positive while 4 (13.3%) were RhD negative. This suggests that over 13% of the pregnant women attending antenatal care will untenably receive anti-D immunoprophylaxis if fetal RhD genotype is not determined. Though the number of patients associated with this proportion is low given the limited sample size of this study, considering the increasing number of pregnancies in developing countries [26, 27], this number could be potentially high. Thus, putting measures in place to abate the unessential administration of anti-D immunoprophylaxis will be of tremendous benefit to both the mother and her unborn baby.

In assessing the expediency of conventional PCR for prenatal sex and RhD genotyping for use in resource-limited facilities, we found that neonates of RhD− pregnant women with RhD+ partners can be genotyped with an excellent level of accuracy (100%) for SRY, RhD exons 5 and 7 but not RhD 10 which showed a 93.3% accuracy. Specifically, comparing prenatal evaluations with postnatal assessments, fetal RhD exons 5 and 7 genotyping from maternal plasma with a median gestational age of 33.0 weeks showed a 100% concordance with neonatal RhD phenotypes after delivery. This finding is consistent with a study by Minon et al. [6] who found that by combining amplification of three exons, the concordance rate of fetal RhD genotypes in maternal plasma and newborn D phenotypes at delivery was 100% (99.8% including one unusual false-positive) based on qPCR. Also in harmony with our study findings are the studies by Lo et al. [15] and Hromadnikova et al. [28]. Evidence suggests that, in order to avoid false-negative results, at least two exons of RHD need to be assessed [20, 29]. Thus, according to the findings of this study, conventional PCR employing the SRY, RhD exons 5 and 7 may be valuable for genotyping fetal sex and RhD from maternal peripheral blood in resource-limited settings which do not possess the sophisticated real-time PCR. A recent study by Mahdavi et al. also confirms the usefulness of the SRY gene for fetal sex determination [19].

The major limitation of this study is that we did not perform qPCR in tandem with the conventional PCR for comparison. However, this limitation is attenuated due to the fact that the prospective nature of the study sex and fetal RhD status were confirmed after the babies were born. Furthermore, the study involved a relatively small sample size. We thus recommend the use of a larger sample size in future studies.

## 5. Conclusion

Our study suggests that conventional PCR using the SRY, RhD exons 5 and 7 could be useful for predicting fetal sex and RhD from maternal peripheral blood in resource-limited settings.[30]

## Figures and Tables

**Table 1:** Demographic and obstetric characteristics of the study participants.

Variable	Frequency (*n* = 30)	Percentage (%)
Maternal age (years)	28.0 (25.0–31.0)^*∗*^	
<25	5	16.7
25–30	17	56.7
31–35	8	26.7
>35		

Gestational age (weeks)	33.0 (12.0–39.0)^*∗*^	
First	2	6.7
Second	4	13.3
Third	24	80
Parity	1 (0–2)^*∗*^	
Gravidity	2 (1–3)^*∗*^	

Marital status		
Married	22	73.3
Single	8	26.7

Educational status		
No formal education	1	3.3
Basic	7	23.3
Secondary	16	53.3
Tertiary	6	20

^*∗*^Data are presented as median (interquartile range).

**Table 2:** Fetal sex, RhD genotype from maternal plasma, and their confirmation after delivery.

Patient ID	Fetal genotype from maternal blood				Neonatal RhD phenotype after delivery	Confirmed sex of neonates
	RHD 5	RHD 7	RHD 10	SRY		
1	+	+	+	−	+	Female
2	+	+	+	−	+	Female
3	+	+	−	+	+	Male
4	+	+	+	−	+	Female
5	−	−	−	−	−	Female
6	+	+	+	−	+	Female
7	+	+	+	+	+	Male
8	+	+	+	+	+	Male
9	+	+	+	−	+	Female
10	+	+	+	−	+	Female
11	−	−	−	−	−	Female
12	+	+	+	−	+	Female
13	+	+	+	+	+	Male
14	+	+	+	−	+	Female
15	+	+	+	−	+	Female
16	+	+	+	−	+	Female
17	+	+	+	−	+	Female
18	−	−	−	+	−	Male
19	+	+	+	−	+	Female
20	+	+	+	+	+	Male
21	+	+	+	+	+	Male
22	+	+	−	−	+	Female
23	+	+	+	−	+	Female
24	+	+	+	−	+	Female
25	+	+	+	−	+	Female
26	+	+	+	+	+	Male
27	−	−	−	−	−	Female
28	+	+	+	+	+	Male
29	+	+	+	−	+	Female
30	+	+	+	−	+	Female

+; positive, −; negative.

**Table 3:** Analytical and diagnostic measures between prenatal RhD and sex genotyping and postnatal measures.

Genotyping	Neonatal RhD phenotype		Concordance	Cohen's kappa	Accuracy	*P* value
	RhD+	RhD−				
Fetal genotype						
RhD5			100.0%	100.0%	100.0%	<0.0001
Positive	26 (100.0)	0 (0.0)				
Negative	0 (0.0)	4 (100.0)				
RhD7			100.00%	100.0%	100.0%	<0.0001
Positive	26 (100.0)	0 (0.0)				
Negative	0 (0.0)	4 (100.0)				
RhD10			92.30%	76.2%	93.3%	0.001
Positive	24 (92.3)	0 (0.0)				
Negative	2 (7.7)	4 (100.0)				

Fetal gender	Neonatal gender					

SRY	Male	Female	100%	100.00%	100.00%	<0.0001
Positive	9 (100.0)	0 (0.0)				
Negative	0 (0.0)	21 (100.0)				

## Data Availability

All relevant data are within the article (and its supplementary information files).

## Supplementary Materials

Supplementary Materials The primers for SYR, RhD exons 5, 7, and 10 used in this study (table S1), graph of DNA purity (figure S1), and yield and agarose gel electrophoresis as visualized under ultraviolet light are given (figure S2).
