# Supplementary material for: Fetal Rhesus D Genotyping and Sex Determination from Maternal Plasma of Rhesus D-Negative Antenatal Population: The Usefulness of Conventional Polymerase Chain Reaction in Resource-limited Settings
Source: Obstet Gynecol Int. 2020 Oct 16;2020:4913793. doi: 10.1155/2020/4913793 (PMC7585667; doi:10.1155/2020/4913793)
Supplement: Supplementary Materials — The primers for SYR, RhD exons 5, 7, and 10 used in this study (table S1), graph of DNA purity (figure S1), and yield and agarose gel electrophoresis as visualized under ultraviolet light are given (figure S2). [file 4913793.f1.docx]

**Table S1: The primers for SRY, RhD exon 5,7 and 10 used in this study.**

| Target gene | Primer | Sequence (5'-3') | Product size |
| --- | --- | --- | --- |
| SRY | Forward | TGGCGATTAAGTCAAATTCGC | 137 bp |
|  | Reverse | CCCCCTAGTACCCTGACAATGTATT |  |
| Exon 5 | Forward | CGCCCTCTTCTTGTGGATG | 82 bp |
|  | Reverse | GAACACGGCATTCTTCCTTTC |  |
| Exon 7 | Forward | CTCCATCATGGGCTACAA | 90 bp |
|  | Reverse | CCGGCTCCGACGGTATC |  |
| Exon 10 | Forward | CCTCTCACTGTTGCCTGCATT | 74 bp |
|  | Reverse | AGTGCCTGCGCGAACATT |  |


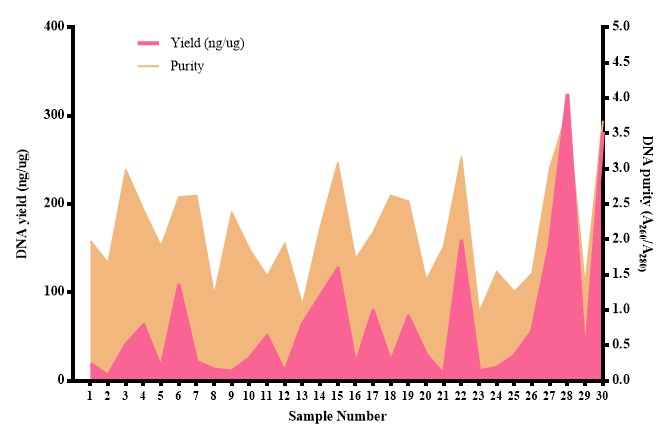


**Figure S1: Graph of DNA purity and yield.**


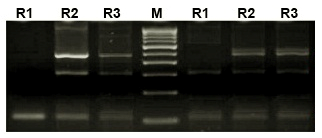


**Figure S2: Agarose gel electrophoresis as visualized under ultraviolet light.**
